# A Trust-Based Methodology to Evaluate Deep Learning Models for Automatic Diagnosis of Ocular Toxoplasmosis from Fundus Images

**DOI:** 10.3390/diagnostics11111951

**Published:** 2021-10-21

**Authors:** Rodrigo Parra, Verena Ojeda, Jose Luis Vázquez Noguera, Miguel García-Torres, Julio César Mello-Román, Cynthia Villalba, Jacques Facon, Federico Divina, Olivia Cardozo, Verónica Elisa Castillo, Ingrid Castro Matto

**Affiliations:** 1 Centro de Investigación, Universidad Americana, Avenida Brasilia 1100, Asunción 1206, Paraguay; verena.ojeda@ua.edu.py (V.O.); jose.vazquez@ua.edu.py (J.L.V.N.); julio.mello@ua.edu.py (J.C.M.-R.); 2 Data Science and Big Data Lab., Universidad Pablo de Olavide, ES-41013 Seville, Spain; mgarciat@upo.es (M.G.-T.); fdivina@upo.es (F.D.); 3 Facultad Politécnica, Universidad Nacional de Asunción, San Lorenzo 2169, Paraguay; cvillalba@pol.una.py; 4 Department of Computer and Electronics, Universidade Federal do Espírito Santo, São Mateus 29932-540, Brazil; jacques.facon@ufes.br; 5 Department of Ophthalmology, Hospital General Pediátrico Niños de Acosta Ñu, San Lorenzo 2169, Paraguay; serviciodeoftalmologiahgp@gmail.com; 6 Departamento de Retina, Cátedra de Oftalmología, Hospital de Clínicas, Facultad de Ciencias Médicas, Universidad Nacional de Asunción, San Lorenzo 2169, Paraguay; vcastillo@med.una.py (V.E.C.); incamatt@hotmail.com (I.C.M.)

**Keywords:** deep learning, ocular toxoplasmosis, machine learning interpretability, trust

## Abstract

In the automatic diagnosis of ocular toxoplasmosis (OT), Deep Learning (DL) has arisen as a powerful and promising approach for diagnosis. However, despite the good performance of the models, decision rules should be interpretable to elicit trust from the medical community. Therefore, the development of an evaluation methodology to assess DL models based on interpretability methods is a challenging task that is necessary to extend the use of AI among clinicians. In this work, we propose a novel methodology to quantify the similarity between the decision rules used by a DL model and an ophthalmologist, based on the assumption that doctors are more likely to trust a prediction that was based on decision rules they can understand. Given an eye fundus image with OT, the proposed methodology compares the segmentation mask of OT lesions labeled by an ophthalmologist with the attribution matrix produced by interpretability methods. Furthermore, an open dataset that includes the eye fundus images and the segmentation masks is shared with the community. The proposal was tested on three different DL architectures. The results suggest that complex models tend to perform worse in terms of likelihood to be trusted while achieving better results in sensitivity and specificity.

## 1. Introduction

Over a third of the world’s human population is exposed to *Toxoplasma gondii*, making Toxoplasmosis one of the most common parasitic diseases worldwide [1]. Ocular toxoplasmosis (OT) occurs if the parasite reaches the retina, as it can damage host cells and neighboring cells leaving primary lesions. OT requires drug-based therapy to eliminate the parasite and the inflammation caused by it. If not treated properly, OT can lead to loss of vision [2].

Ophthalmologists conduct eye exams that look for lesions caused by the disease in eye fundus images to diagnose OT. Clinical manifestations of the disease tend to be highly characteristic; however, atypical manifestations can cause false-negative errors even by experienced doctors. Clinical examination is considered the diagnostic standard, due to the lack of a sufficiently sensitive lab test [3].

Machine learning is a subfield of artificial intelligence that allows computers to learn from existing data and make predictions. Its application has improved the performance of many challenging tasks in medical imaging, with a considerable impact on ophthalmology based on fundus photography, optical coherence tomography and slit-lamp imaging [4].

Deep learning (DL) is a subfield of machine learning based on artificial neural networks (ANN), a paradigm inspired by the human brain. DL models allow end-to-end learning, skipping the feature engineering step that was required by traditional computer vision approaches [5]. DL models have achieved promising results in automatic classification of images, and they have brought breakthroughs to the state of the art in recent years [6].

In particular, when applied to retinal images for medical diagnosis and prognosis, convolutional neural networks (CNNs) have been able to identify and estimate the severity of ocular diseases, such as age-related macular degeneration [7] and diabetic retinopathy [8,9]. Moreover, models have been trained to detect lesions caused by these diseases and classify them according to their severity [10].

Hasanreisoglu et al. [11] explored similar techniques for OT diagnosis using fundus images. Parra et al. [12] attempted an additional network architecture and achieved promising results, in addition to publishing an open OT dataset. To the best of our knowledge, these are the only works that have applied deep learning to OT diagnosis.

In the field, most works have been focused on the predictive power of the model. However, despite the good results obtained, the medical community is skeptical about its use due, mainly, to the difficulty in the interpretation of the results. Human factors play an important role in the diagnosis, and they must be taken into account to increase the reliability of the models induced and to extend human-AI collaboration. The concept of *Trust* arises in this context, defined as the intention to accept vulnerability based on positive expectations [13]. Currently, a lack of trust in AI systems is a significant drawback in the adoption of this technology in healthcare [14]. Understanding the reasons behind predictions, and analyzing them considering prior knowledge about the application domain, can be important to establish trust [15].

Zhang et al. defined interpretability as the ability to provide explanations in understandable terms to a human [16]. As such, interpretability methods can be used to obtain an explanation of the output of a predictive model. Attribution methods, a family of interpretability methods, assign credit (or blame) with regards to the prediction to the input features. For images, this means that they assign a score to each of the input pixels.

Several deep learning attribution methods are based on gradients, i.e., partial derivatives of the output with respect to the input. Gradient * Input [17], Integrated Gradients [18], Layer-wise Relevance Propagation (LRP) [19] and DeepLIFT [20] are examples of such methods. Although they use gradients differently to compute attribution scores, Ancona et al. have shown these methods to be strongly related, if not equivalent under certain conditions [21].

Attribution methods have been applied to classification problems with retinal images, to enrich predictions presented to physicians. Sayres et al. explored integrated gradients to grade diabetic retinopathy [22], and Mehta et al. used the same method for automatic detection of glaucoma [23].

A general-purpose trust metric was proposed by Wong et al. [24] and extended by Hryniowski et al. [25]. They were experimentally tested with Imagenet with insightful results. Interpretability, a prerequisite of trust, is known to be a domain-specific notion [26]. Hence, we argue that domain-specific trust metrics are important for machine learning adoption.

In this study, we propose a method to quantitatively evaluate the trustworthiness of a model in the OT diagnosis domain. We do this by comparing the average attribution scores of pixels that belong to a lesion vs. the rest of the pixels. We assume that doctors are more likely to trust a model if its predictions are based on the features they consider for their diagnosis. Hence pixels within lesions should have higher attribution scores than the rest for an OT model to be considered trustworthy.

The rest of this paper is organized as follows. Section 2 introduces the main concepts of this work, including the data used. Then, in Section 3, the experimental results are described. The discussion about such results is given in Section 4. Finally, Section 5 presents the conclusions of this work.

## 2. Materials and Methods

In this section, the main characteristics of the data are first presented. Then, the different Deep Learning architectures are introduced. Finally, the proposed evaluation methods are described.

### 2.1. Dataset

**Figure 1:**
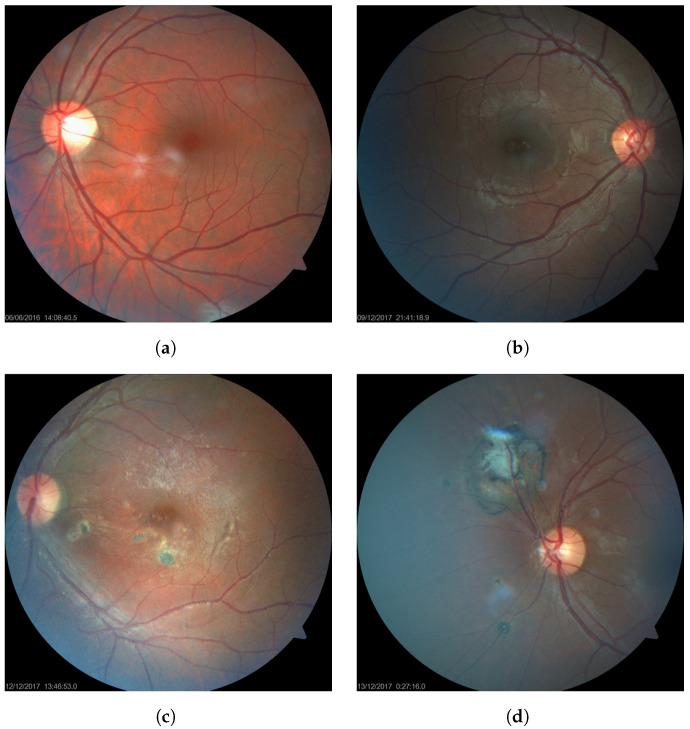
A sample of healthy (**a**,**b**) and unhealthy (**c**,**d**) retinal fundus images from the dataset.

Predictive models were trained and evaluated on a dataset of 160 eye fundus images. These images were collected at the Hospital de Clínicas in Asunción (Paraguay) by members of the Department of Ophthalmology. Some examples of the dataset can be seen in Figure 1. The complete dataset can be found online and is freely available for research purposes.

Images were captured using a Zeiss brand camera, model Visucam 500, operated by experienced ophthalmologists. Each image was manually segmented by an ophthalmologist using an open source labeling tool (https://labelstud.io (accessed on Wednesday, 20 October 2021)) to manually highlight OT entities (active lesions and inactive scars).

**Figure 2:**
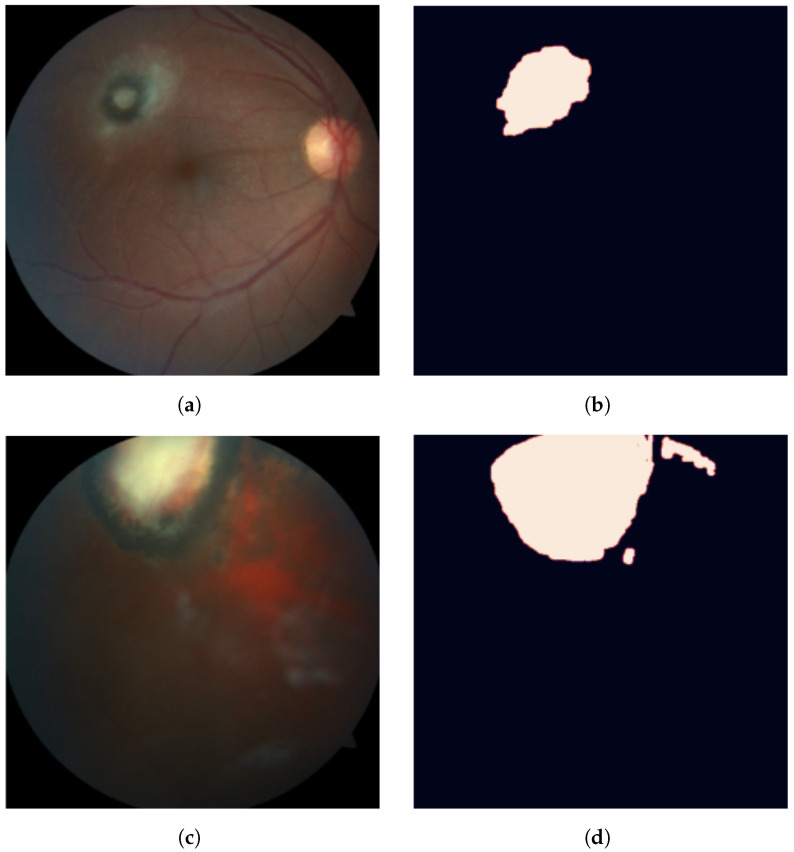
A sample of unhealthy eye fundus images (**a**,**c**) with their corresponding masks of segmented OT lesions (**b**,**d**) from the dataset.

Active lesions have variable size, white or yellow color, blurry edges and a cottony center. They might be associated with a brown retinal hyperpigmentation area, which is compatible with previous scar lesions. In some cases, active lesions can be hard to differentiate due to the presence of vitreitis. Inactive lesions have variable size with possible brown hyperpigmentation, with a stunted yellow or white center. An example of these annotations can be seen in Figure 2.

### 2.2. Model Training

Deep learning models and, in particular, CNNs, have achieved state-of-the-art results in terms of predictive power for computer vision use cases [27]. Convolutional neural networks are a particular type of feedforward neural networks (artificial neural networks with no backlinks) that is normally composed of a combination of layers:

- Convolutional layers: capture local features by sliding a set of kernels over their input.
- Pooling layers: are used to downsample the output of convolutional layers.
- Fully-connected layers: are often used as the final layers of the model, to perform the final prediction.

As kernels share weights with all neurons, they help significantly in reducing the total number of parameters of the network. Thus, CNNs allow building neural networks with many layers with fewer parameters than other architectures [28].

We evaluate three different architectures:

- A CNN model with a few convolutional layers initialized with random weights.
- A VGG16 [29] model pretrained on the Imagenet dataset.
- A Resnet18 [30] model pretrained on the Imagenet dataset.

VGG16 is an architecture proposed by Simonyan and Zisserman, which was the first to experiment with smaller kernel sizes achieving promising results and increased depth of the model. Furthermore, Resnet18, which introduced the concept of residual connections. Residual connections help transfer knowledge from previous layers, alleviating the vanishing gradient problem that neural networks often suffer from. Residual networks allowed even deeper models to be trained, with a decreased number of parameters [28].

A comparison of the three architectures in terms of number of parameters and depth is shown in Table 1.

**Figure 3:**
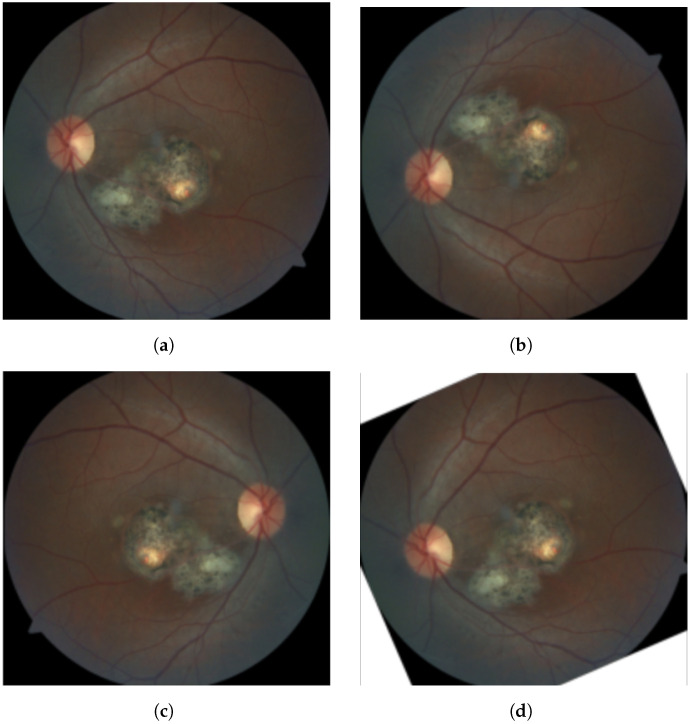
An original eye fundus training image (**a**) with some example transformations, such as vertical flip (**b**), horizontal flip (**c**) and rotation (**d**), which are computed for data augmentation.

**Figure 4:**
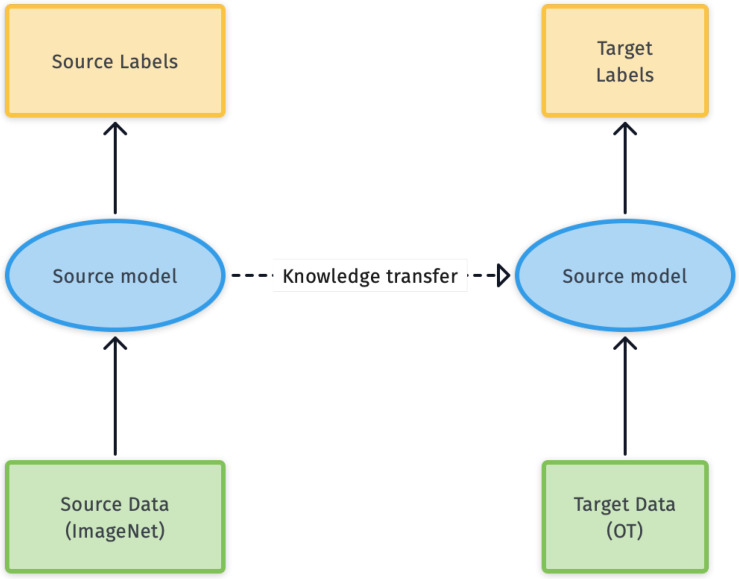
A schematic overview of transfer learning.

Data augmentation based on random flips and crops was performed for all models, as shown in Figure 3. The last two models leverage transfer learning, i.e., they were pretrained on a larger general-purpose image dataset and then, with minor modifications to the learned weights, applied to OT classification for which less data is available. This is common when applying DL in domains where it is very difficult to build well-annotated datasets on a large scale due to the cost of acquiring data and annotations [31]. The idea of transfer learning is represented graphically in Figure 4.

Models were optimized for 50 epochs using stochastic gradient descent (SGD) with a batch size of 32. Binary cross-entropy loss was used as the optimization target. The dataset was split into training (70%), validation (10%) and test (20%) sets. The training set was used for model fitting, the validation set for hyperparameter tuning and the test set to make the final model evaluation.

### 2.3. Model Evaluation

All models were evaluated using traditional predictive performance metrics: accuracy, sensitivity and specificity. In addition to that, we propose a method to obtain a trust score based on feature attributions, which is described in detail below. We only consider ed images with lesions that were correctly classified by the models (as a reminder, we consider an eye fundus image to be unhealthy if there are any lesions) for our evaluation, since our analysis depends on OT entities that were segmented by ophthalmologists.

#### 2.3.1. Measuring Feature Importance: Pixel Attribution Scores

Attribution methods provide scores for each of the input features that estimate the relevance they had on the prediction. Formally, given a deep neural network (DNN) $F:R^{n}\to \left[0,1\right]$, let $x\in R^{n}$ be the model input. An attribution method can be seen as a function $A\left(F,x\right)=\left[s_{1},\ldots ,s_{n}\right]$, where $s_{1},\ldots ,s_{n}$ are referred to as *attribution scores*. In this study, we use Integrated Gradients (IG) as the attribution method of choice.

Let $x^{\prime }\in R^{n}$ be a baseline input of the model, which is usually a black image for image networks. Integrated gradients are defined as the integral of the gradients along the path from the baseline $x^{\prime }$ to the input *x*. The integrated gradient for the *i*th dimension is defined as follows:

$$IntegratedGradients_{i}\left(x\right)::=\left(x_{i}-x_{i}^{{}^{\prime }}\right)\times \int_{\alpha =0}^{1}\frac{\partial F(x^{{}^{\prime }}+\alpha \times \left(x-x^{{}^{\prime }}\right))}{\partial x_{i}}d\alpha$$

where $\frac{\partial F(x)}{\partial x_{i}}$ is the gradient of F(x) along the *i*th dimension and α is an interpolation constant to perturb features by.

We can calculate an attribution score per feature using IG. To obtain a per-pixel attribution score, we sum scores across RGB channels. The proposal of this study is independent of the actual attribution method selected.

#### 2.3.2. Evaluating a Prediction: To Trust or Not to Trust?

Given a particular pixel attribution matrix $A\in R^{n}$ and a mask of OT entities for the original image, some pixels belong to an OT entity and others do not. Assume that those two groups of pixels were sampled from different populations, *L* and *R*. We expect the median of *L* to be larger than that of *R* for OT cases, i.e., pixels from the lesions identified by a physician should be relatively more relevant for the model to elicit trust from them. We can test this hypothesis by using a one-sided Mann–Whitney U test such that:

$h_{0}$: The median of *R* is larger or equal than the median of *L*.

$h_{1}$: The median of *L* is larger than the median of *R*.

Therefore, we can define a binary trust function *t* as:

$$t\left(A\right)=\left\{\begin{array}{cc} 0, & \mathrm{if}\;\mathrm{the}\;p-\mathrm{value}\;<\;0.05,\;i.e.,\;\mathrm{we}\;\mathrm{fail}\;\mathrm{to}\;\mathrm{reject}\;\mathrm{the}\;\mathrm{null}\;\mathrm{hypothesis}\\ 1, & \mathrm{otherwise} \end{array}\right.$$

#### 2.3.3. Evaluating a Model Given a Dataset: Aggregating Our Results

Given a test set of images, a model is scored by calculating the ratio of images for which we obtain a one after applying *t* to their pixel-attribution matrix. This aggregate represents the proportion of images for which to model is likely to be considered trustworthy by an ophthalmologist.

The general purpose trust score proposed by Wong et al. [24] and extended by Hryniowski et al. [25] defines trust based on the answer to two questions: (1) How much trust do we have in a model that gives wrong answers with great confidence? and (2) How much trust do we have in a model that gives right answers hesitantly? However, valuable, interpretability and trust are known to be domain-specific notions [26]. Hence, the trust score proposed in this work incorporates domain-specific knowledge (masks) and compares it with the attribution matrix to answer the question: Did the model consider the features that an ophthalmologist would have taken into account (lesions) for this prediction?

**Figure 5:**
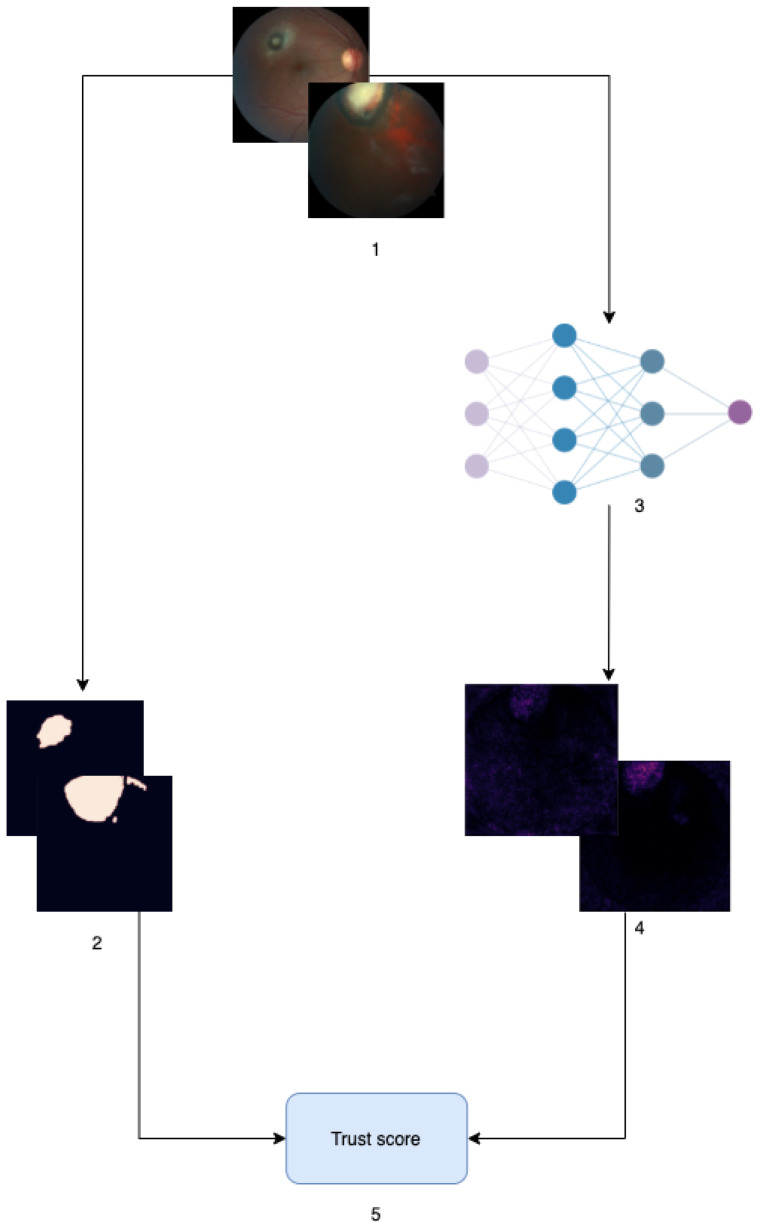
A schematic overview of the general process for trust evaluation.

A general overview of the process to evaluate a model is depicted in Figure 5 and can be summarized as follows: (i) an eye fundus dataset was collected by ophthalmologists at the Hospital de Clínicas of Asunción, Paraguay, (ii) physicians manually segmented OT entities for every image that had lesions, (iii) a predictive model is trained on a subset of the eye fundus dataset, (iv) pixel- attribution matrices are computed for all correctly-predicted sick images of a test set and, finally, (v) segmentation masks and attribution matrices are compared using a Mann–Whitney U test, and the results are aggregated to calculate the model trust score.

## 3. Results

The experiments were performed on a Google Colab Pro account, which provides Nvidia T4 and P100 graphic cards and up to 25 GB of RAM. The models were implemented using Pytorch 1.4. Models were trained with a batch size of 32, a learning rate of 1 × 10${}^{-2}$ and stochastic gradient descent (SGD) as the optimizer, and these hyperparameters were selected according to the selection process performed by Parra et al. [12].

Two experiments were performed:

- Models were trained and evaluated with respect to accuracy, sensitivity and specificity, to contrast them with the results of the proposed trust metric and then,
- Models were evaluated using the proposed trust score on all correctly-predicted sick images from the test set.

### 3.1. Common Predictive Metrics

After fine-tuning all predictive models common metrics used to evaluate predictive power were computed on the complete test set. Table 2 summarizes the results in terms of accuracy, sensitivity and specificity. The goal of this experiment was to determine if the evaluated models ranked similarly to comparisons made in other domains. As expected, both VGG and Resnet achieve better results than the vanilla CNN. Interestingly, better results were achieved with VGG than with Resnet as opposed to the results published for ImageNet [30].

### 3.2. Trust Score

**Figure 6:**
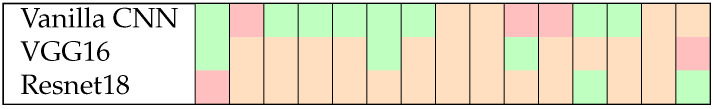
Visualizing results: each cell represents an image of the test set that was predicted using a model. Green cells represent correct and trustworthy predictions (i.e., those where the lesions were relevant for the model output); orange cells are those where the model predicted the right label, but the prediction might not be trustworthy; and red cells are prediction errors.

The proposed trust score was calculated for each of the models on the subset of correctly-labeled images from the test set, as depicted in Section 5. Aggregated results for each the compared models are summarized in Table 3. Predictive metrics are included to better contrast their relationship to the proposed score. The results show that models that scored higher in terms of traditional metrics associated with predictive power, e.g., accuracy, sensitivity and specificity performed worse in terms of the proposed trust score. This can be seen on a per-image basis in Figure 6. In addition to this, numeric values associated with the trust score calculation on a per-image basis can be found in Table A1 of Appendix A.

## 4. Discussion

**Figure 7:**
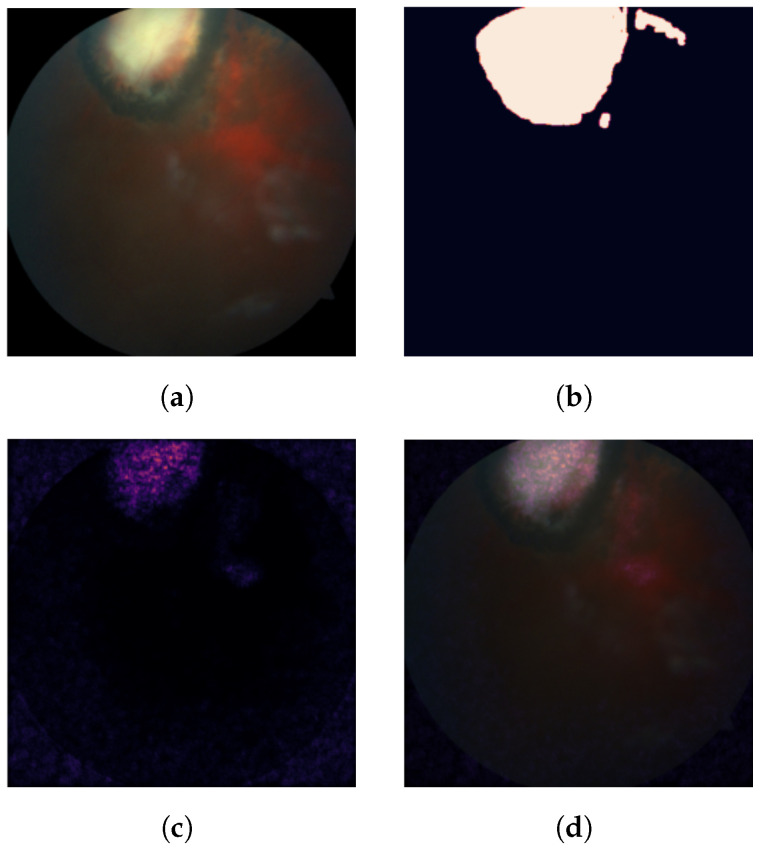
An example of an unhealthy eye fundus image that was correctly classified by the CNN model (**a**), the mask segmented by an ophthalmologist (**b**), a heatmap of the IG-based pixel attribution scores (**c**) and the attribution scores as an overlay (**d**). Median pixel attribution score differences were statistically significant between lesion and non-lesion areas.

**Figure 8:**
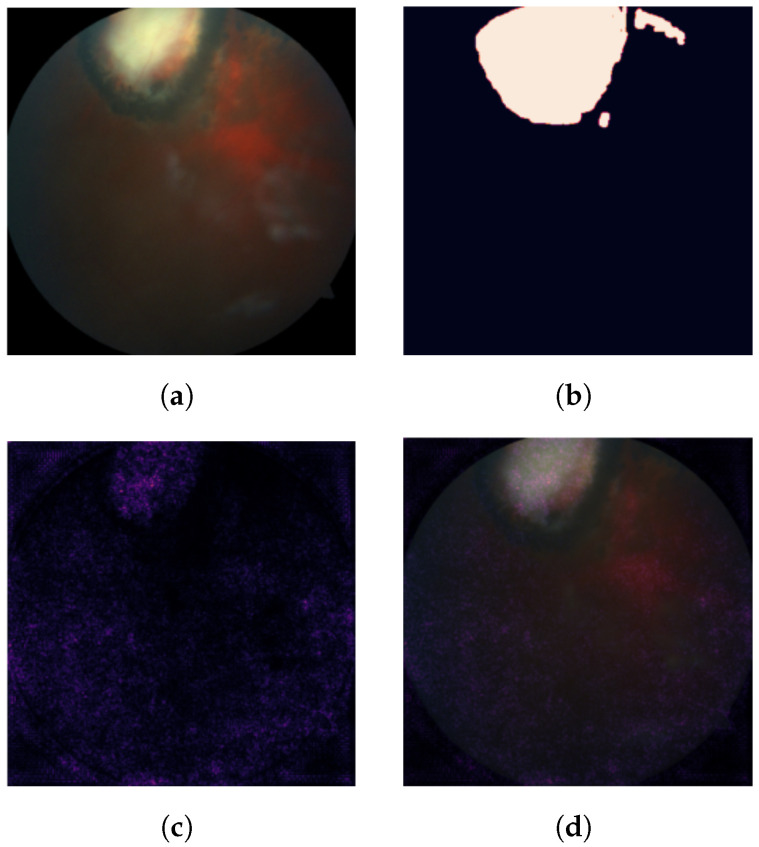
An example of an unhealthy eye fundus image that was correctly classified by the Resnet18 model (**a**), the mask segmented by an ophthalmologist (**b**), a heatmap of the IG-based pixel attribution scores (**c**) and the attribution scores as an overlay (**d**). Median pixel attribution score differences were not statistically significant between lesion and non-lesion areas.

Exploratory analysis of the IG attribution maps confirms the intuition behind the proposed trust score. Figure 7 shows an example prediction for which the model was considered trustworthy. This can be visually verified as the attribution scores are clustered around the area of the lesion. Figure 8 shows an example prediction for which the model was considered untrustworthy. One can visually confirm that pixel attribution scores are scattered and less concentrated on the lesion area.

The obtained results suggest that predictions made by the most accurate deep learning might be harder to trust by experienced physicians. These findings agree with the existing literature, as it is known that healthcare workers often find it challenging to trust complex machine-learning models [32].

Interestingly, the relationship between the trust score and the number of parameters of the trained models (a common proxy for complexity) is not perfectly inverse. Although it is clear that the simple CNN scored much higher, the trust score for VGG16 was higher than that of Resnet18, despite having approximately 10-times more trainable parameters. This suggests that further research is needed regarding what exactly is it about complexity that punishes trustworthiness of the predictions, e.g., Are residual blocks bad for model trust? In other words, Can the key architectural decisions that lead to poor trustworthiness be identified?

Answering the previous question can lead to developing better building blocks for DL and machine learning in general, and this represents a needed, but challenging, change in the way state-of-the-art models are currently evaluated. Considering metrics beyond performance power is key to achieving mainstream adoption of predictive models in the healthcare domain.

## 5. Conclusions

We evaluated three different DL architectures and observed an inverse relation between the predictive power and our trust score. These results suggest that trust should also be considered for model selection, in addition to more traditional metrics, such as sensitivity and specificity. This is particularly the case if we expect deep learning models to be adopted by the medical community.

The main contributions of this work are: (i) an open dataset of annotated eye fundus images for OT diagnosis and (ii) a domain-specific method to evaluate predictive models with respect to trust (i.e., how likely a physician is to trust a model’s predictions) for OT diagnosis.

Extensions to our work can include: (i) a user study with ophthalmologists could help validate that our trust score adequately models their reactions to different model predictions, (ii) comparing the results using alternative attribution methods and (iii) comparing our score with traditional ML models by using an extension of IG that supports non-differentiable models [33].

## Figures and Tables

**Table 1:** A comparison of the three selected deep learning models.

Model	Parameters (Millions)	Layers
Vanilla CNN	5.6	6
VGG16	138	16
Resnet18	11	152

**Table 2:** Predictive metrics comparison for the three deep learning models.

Model	Accuracy	Sensitivity	Specificity
Vanilla CNN	0.75	0.75	0.75
VGG16	0.96875	1.0	0.9375
Resnet18	0.9375	0.9375	0.9375

**Table 3:** Metric comparison including trust score for the three deep learning models.

Model	Accuracy	Sensitivity	Specificity	Trust
Vanilla CNN	0.75	0.75	0.75	0.67
VGG16	0.96875	1.0	0.9375	0.21
Resnet18	0.9375	0.9375	0.9375	0.14

## Data Availability

The data used to conduct this work is available at https://doi.org/10.5281/zenodo.4479724 (accessed on Wednesday, 20 October 2021).

## Abbreviations

The following abbreviations are used in this manuscript:

AI	Artificial Intelligence
DL	Deep Learning
OT	Ocular Toxoplamosis
CNN	Convolutional Neural Network
IG	Integrated Gradients

## Appendix A. Detailed Results

**Table A1:** Results per pair of model and test image. Images are identified by a unique code (IID). Results include the mean attribution score for lesion-related and non-lesion-related pixels and the *p*-value obtained for the Mann–Whitney test ^1^.

IID	Vanilla CNN	VGG16	Resnet18
81	Mean lesion attribution: 0.00034721767224766176 Mean non-lesion attribution: −6.601 419 281 104 99 × 10^−6^ Mann–Whitney *p*-value: 0.0	Mean lesion attribution: 0.0023451965695009515 Mean non-lesion attribution: −1.505 774 503 219 224 6 × 10^−5^ Mann–Whitney *p*-value: 2.182 831 996 769 315 × 10^−7^	Mean lesion attribution: −6.291 439 449 744 539 × 10^−5^ Mean non-lesion attribution: 4.173 565 166 532 544 × 10^−6^ Mann–Whitney *p*-value: 0.8461449179567923
156	misclassified	Mean lesion attribution: 7.608 095 877 901 965 × 10^−5^ Mean non-lesion attribution: 9.139 120 141 825 367 × 10^−6^ Mann–Whitney *p*-value: 0.8894574555554062	Mean lesion attribution: 1.026 627 135 195 600 7 × 10^−5^ Mean non-lesion attribution: 1.283 862 080 623 880 5 × 10^−5^ Mann–Whitney *p*-value: 0.5119256881011157
148	Mean lesion attribution: 6.443 540 099 421 086 × 10^−5^ Mean non-lesion attribution: −3.871 953 049 444 241 × 10^−6^ Mann–Whitney *p*-value: 0.0	Mean lesion attribution: 0.0004341474669653702 Mean non-lesion attribution: −2.815 535 276 382 608 5 × 10^−5^ Mann–Whitney *p*-value: 0.12678531658770215	misclassified
144	Mean lesion attribution: 2.284 996 167 701 541 6 × 10^−5^ Mean non-lesion attribution: −1.224 208 807 727 668 7 × 10^−7^ Mann–Whitney *p*-value: 2.1783883659119423 × 10^−143^	Mean lesion attribution: 9.005 965 358 771 226 × 10^−5^ Mean non-lesion attribution: 7.268116949538503 × 10^−6^ Mann–Whitney *p*-value: 0.2580941242701976	Mean lesion attribution: 2.337 102 520 230 451 4 × 10^−5^ Mean non-lesion attribution: 1.267864040454379 × 10^−5^ Mann–Whitney *p*-value: 0.9355251360530437
97	Mean lesion attribution: 6.336 514 502 731 98 × 10^−5^ Mean non-lesion attribution: 1.0295273698471493 × 10^−6^ Mann–Whitney *p*-value: 0.0	Mean lesion attribution: 0.00031431523156589153 Mean non-lesion attribution: −9.295 814 133 027 072 × 10^−7^ Mann–Whitney *p*-value: 0.4886499718139443	Mean lesion attribution: 8.424 714 848 856 002 × 10^−6^ Mean non-lesion attribution: 1.2007089850336847 × 10^−5^ Mann–Whitney *p*-value: 0.976338912018821
151	Mean lesion attribution: 8.273 648 382 325 341 × 10^−5^ Mean non-lesion attribution: −1.5860174859485157 × 10^−6^ Mann–Whitney *p*-value: 0.0	Mean lesion attribution: 0.00021355240374255574 Mean non-lesion attribution: 4.000 040 678 326 502 × 10^−6^ Mann–Whitney *p*-value: 0.03181811783986231	Mean lesion attribution: 5.638 891 517 729 174 × 10^−5^ Mean non-lesion attribution: 6.625146340744238 × 10^−6^ Mann–Whitney *p*-value: 0.40842097615151357
142	Mean lesion attribution: 9.391 440 979 266 882 × 10^−5^ Mean non-lesion attribution: 5.0270287766254336 × 10^−6^ Mann–Whitney *p*-value: 4.48215423628502 × 10^−230^	Mean lesion attribution: 0.0008307758169506642 Mean non-lesion attribution: 1.188 879 169 016 227 2 × 10^−5^ Mann–Whitney *p*-value: 0.11516303087912255	Mean lesion attribution: 0.00015584305846376865 Mean non-lesion attribution: 6.200 042 851 696 207 × 10^−6^ Mann–Whitney *p*-value: 0.3019700368885526
118	Mean lesion attribution: −2.179 597 111 096 857 7 × 10^−5^ Mean non-lesion attribution: 1.2655909104444177 × 10^−5^ Mann–Whitney *p*-value: 1.0	Mean lesion attribution: 0.00016869457829025018 Mean non-lesion attribution: 7.862 114 094 452 912 × 10^−6^ Mann–Whitney *p*-value: 0.7708368497428252	Mean lesion attribution: 9.424 270 874 506 554 × 10^−5^ Mean non-lesion attribution: 3.1994922104513376 × 10^−7^ Mann–Whitney *p*-value: 0.6965993050535022
150	Mean lesion attribution: −7.918 849 021 031 336 × 10^−5^ Mean non-lesion attribution: 1.583 111 698 817 516 7 × 10^−5^ Mann–Whitney *p*-value: 1.0	Mean lesion attribution: 0.00013991786062732565 Mean non-lesion attribution: 9.315 094 828 690 027 × 10^−6^ Mann–Whitney *p*-value: 0.6484420024657709	Mean lesion attribution: 1.135 622 422 691 986 × 10^−5^ Mean non-lesion attribution: 1.0066499735435533 × 10^−5^ Mann–Whitney *p*-value: 0.5955185797651998
94	misclassified	Mean lesion attribution: 0.00046278512907457894 Mean non-lesion attribution: −6.857 434 254 485 007 × 10^−6^ Mann–Whitney *p*-value: 1.6871382541385086 × 10^−9^	Mean lesion attribution: 2.319106013366875 × 10^−5^ Mean non-lesion attribution: 1.304 558 028 864 510 6 × 10^−6^ Mann–Whitney *p*-value: 0.7233509758415473
132	misclassified	Mean lesion attribution: 0.0007116612771495348 Mean non-lesion attribution: −5.007 881 843 520 115 × 10^−6^ Mann–Whitney *p*-value: 0.9661658611357002	Mean lesion attribution: 3.984 074 887 705 268 × 10^−6^ Mean non-lesion attribution: 1.8449527437055958 × 10^−7^ Mann–Whitney *p*-value: 0.9659463877638184
146	Mean lesion attribution: 6.460267789192614 × 10^−5^ Mean non-lesion attribution: 5.149 698 277 781 459 5 × 10^−5^ Mann–Whitney *p*-value: 3.629556224709794 × 10^−15^	Mean lesion attribution: 0.00033481917634950995 Mean non-lesion attribution: −5.838 907 669 173 114 × 10^−6^ Mann–Whitney *p*-value: 0.9474421613415959	Mean lesion attribution: 3.984 074 887 705 268 × 10^−6^ Mean non-lesion attribution: 1.8449527437055958 × 10^−7^ Mann–Whitney *p*-value: 0.05547639441097111
99	Mean lesion attribution: 1.0623468091879256 × 10^−5^ Mean non-lesion attribution: −8.381 454 520 194 11 × 10^−7^ Mann–Whitney *p*-value: 8.705342309642405 × 10^−12^	Mean lesion attribution: 0.00011561437034381344 Mean non-lesion attribution: 1.122 893 524 636 988 8 × 10^−6^ Mann–Whitney *p*-value: 0.9991945637023955	Mean lesion attribution: 4.387 994 697 815 6 × 10^−5^ Mean non-lesion attribution: 5.952850761770384 × 10^−6^ Mann–Whitney *p*-value: 0.8881489550295014
117	Mean lesion attribution: −3.639 901 875 997 307 6 × 10^−5^ Mean non-lesion attribution: 1.4487363899890637 × 10^−5^ Mann–Whitney *p*-value: 1.0	Mean lesion attribution: 0.0001403303076260771 Mean non-lesion attribution: 1.199 786 422 310 879 6 × 10^−5^ Mann–Whitney *p*-value: 0.8978942267882748	Mean lesion attribution: 6.2256477107090875 × 10^−6^ Mean non-lesion attribution: 1.240 475 023 789 905 8 × 10^−5^ Mann–Whitney *p*-value: 0.503937509947166
111	Mean lesion attribution: 6.573 892 462 999 344 × 10^−10^ Mean non-lesion attribution: 6.415230420503763 × 10^−6^ Mann–Whitney *p*-value: 0.9986524555283746	misclassified	Mean lesion attribution: 0.00011425205913621127 Mean non-lesion attribution: −2.209 874 594 196 942 × 10^−6^ Mann–Whitney *p*-value: 0.024341820096032026

^1^*p*-values of 0.0 are smaller than the smallest floating point number representable by Numpy.
